# Referral to and enrolment in cardiac rehabilitation after open-heart surgery in the Netherlands

**DOI:** 10.1007/s12471-021-01598-z

**Published:** 2021-07-14

**Authors:** D. Conijn, R. A. F. de Lind van Wijngaarden, H. M. Vermeulen, T. P. M. Vliet Vlieland, J. J. L Meesters

**Affiliations:** 1 Department of Orthopaedics, Rehabilitation and Physical Therapy, Leiden University Medical Center, Leiden, The Netherlands; 2 Physical Therapy Sciences, program in Clinical Health Sciences University Medical Centre Utrecht, Utrecht University, Utrecht, The Netherlands; 3 Department of Cardiothoracic Surgery, Leiden University Medical Center, Leiden, The Netherlands; 4 Department for Innovation, Quality and Research, Basalt Rehabilitation Centre, The Hague/Leiden, The Netherlands

**Keywords:** Cardiac rehabilitation, Referral and consultation, Open-heart surgery

## Abstract

**Aim:**

Although referral to phase 2 cardiac rehabilitation (CR) following open-heart surgery is recommended in professional guidelines, according to the literature, participation rates are suboptimal. This study investigates the referral and enrolment rates, as well as determinants for these rates, for phase 2 CR following open-heart surgery via sternotomy.

**Methods:**

A cross-sectional survey study was conducted among patients who underwent open-heart surgery via sternotomy in a university hospital. Data on referral and enrolment rates and possible factors associated with these rates (age, sex, type of surgery, educational level, living status, employment, income, ethnicity) were collected by a questionnaire or from the patient’s medical file. Univariate logistic regression analysis (odds ratio) was used to study associations of patient characteristics with referral and enrolment rates.

**Results:**

Of the 717 eligible patients, 364 (51%) completed the questionnaire. Their median age was 68 years (interquartile range 61–74) and 82 (23%) were female. Rates for referral to and enrolment in phase 2 CR were 307 (84%) and 315 (87%), respectively. Female sex and older age were independently associated with both non-referral and non-enrolment. Additional factors for non-enrolment were surgery type (coronary artery bypass grafting with valve surgery and miscellaneous types of relatively rare surgery), living alone and below-average income.

**Conclusion:**

Phase 2 CR referral and enrolment rates for patients following open-heart surgery were well over 80%, suggesting adequate adherence to professional guidelines. During consultation, physicians and specialised nurses should pay more attention to certain patient groups (at risk of non-enrolment females and elderly). In addition, in-depth qualitative research to identify reasons for non-referral and/or non-enrolment is needed.

## What’s new?

- Data on referral and enrolment rates for phase 2 cardiac rehabilitation (CR) after open-heart surgery are scarce and suggest that these rates are suboptimal.
- In our cohort, the guidelines on referral to and enrolment in phase 2 CR were well adhered to in our cohort, although some subgroups should be referred more frequently.
- A deeper understanding of motives for patients not enrolling in phase 2 CR is needed to develop proper strategies to improve post-surgery care.

## Introduction

Cardiac surgery via sternotomy, referred to as open-heart surgery, is a common and effective intervention for treating heart disease, e.g. coronary heart disease, heart valve disease or aortic aneurysm [1]. In the Netherlands, 14,937 open-heart surgeries were performed in 2017 [1].

The American College of Cardiology Foundation and the European Society of Cardiology strongly recommend cardiac rehabilitation (CR) after open-heart surgery to restore quality of life and to improve functional capacity [2–5]. In the Netherlands, CR comprises three phases. Phase 1 consists of in-hospital CR, which starts immediately after surgery and lasts until hospital discharge. Phase 2 includes outpatient CR, which usually starts 6 weeks after surgery due to consolidation of the sternum. This phase varies in duration based on severity, treatment goals and local procedures. In severe or special cases, (partial) inpatient phase 2 CR may be indicated. Phase 3 is the post-CR phase, which starts after phase 2 and focusses on achieving or maintaining an active lifestyle [4].

**Fig. 1:**
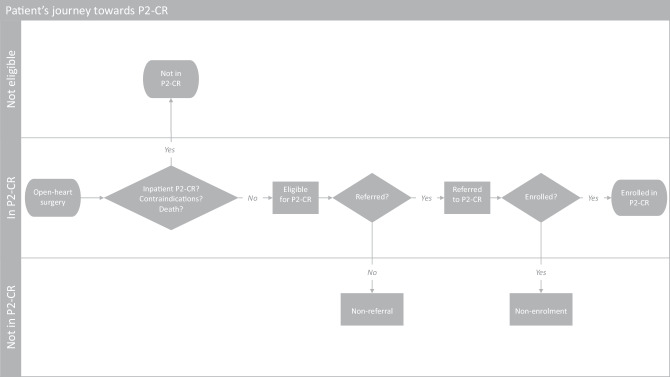
Patient’s journey towards phase 2 cardiac rehabilitation (*P2-CR*) after open-heart surgery in the Netherlands

Phase 2 is the most extensive phase of CR and consists of comprehensive, long-term programmes involving medical evaluation, prescribed exercise, cardiac risk factor modification, education and counselling. These programmes are designed to limit the physiological and psychological effects of cardiac illness, reduce the risk of sudden death or re-infarction, control cardiac symptoms, stabilise the atherosclerotic process, and enhance the psychosocial and vocational status of selected patients [6–8]. The patient’s journey in the Netherlands is guided by the cardiologist who refers the patient to phase 2 CR (Fig. 1). Subsequently, patients are called in for a first screening. Those patients who show up and are admitted, can start phase 2 CR.

Although phase 2 CR after cardiac illness, including open-heart surgery, is effective and is recommended by several (international) guidelines, the actual participation rates of phase 2 CR programmes after cardiac illness vary among countries worldwide (3–90%) [9–11]. Studies on open-heart surgery specifically report that 60–90% of the eligible patients from different European countries enrol in phase 2 CR after surgery [11, 12]. Based on their Dutch cohort study, Van Engen et al., reported in 2013 a phase 2 CR enrolment rate of 59% among Dutch patients after open-heart surgery [13]. This is relatively low compared with other European countries, suggesting that a considerable proportion of Dutch patients may not receive appropriate care after open-heart surgery.

Specific patient characteristics associated with non-referral and non-enrolment have been described in several studies over the last 10 years [9, 10, 14–23]. A review published in 2018 focussed on cardiac illness in general, partly pertaining to patients after open-heart surgery [10]. For that review, Resurrección et al. systematically analysed 43 prospective cohort studies on factors of non-referral to and/or non-enrolment in phase 2 CR [10]. In total, more than 50 intrapersonal, clinical, interpersonal, logistical, CR programme and health system factors of patients with cardiac illness were found to be associated with phase 2 CR non-referral and/or non-enrolment [10]. Articles on this topic published after 2018 did not reveal any additional factors to be associated with non-referral and/or non-enrolment [24, 25].

Although the available literature describes which factors are associated with non-referral to and/or non-enrolment in phase 2 CR for cardiac illness in general, no specific studies on the effect of these factors on phase 2 CR referral and enrolment after open-heart surgery are available. This is relevant since patients start phase 2 CR 6 weeks after surgery due to the aforementioned sternal consolidation. This period is longer than the period of 2–4 weeks after non-open-heart surgery indications and increases the risk of non-referral and/or non-enrolment [4, 26, 27].

Therefore, our aim was to describe the rates of referral to and enrolment in phase 2 CR in patients after open-heart surgery and to explore which factors are associated with phase 2 CR referral and enrolment after open-heart surgery in a Dutch university medical centre.

## Methods

A cross-sectional survey study was conducted at the Leiden University Medical Center (LUMC) in Leiden, the Netherlands. The study protocol was approved by the Medical Ethics Review Committee of the LUMC (P18.234).

### Cardiac rehabilitation in the Netherlands

In the Netherlands, all citizens are legally obliged to have medical insurance coverage. By law, the basic health insurance policy covers all expenses of phase 2 CR following open-heart surgery. According to the Dutch CR guidelines [4], every patient who is eligible for phase 2 CR after open-heart surgery (if physical and mental status permit) should be referred to phase 2 CR by their cardiologist [4, 5].

### Recruitment and selection of participants

All adult patients who underwent open-heart surgery (e.g. valve replacement, coronary artery bypass grafting (CABG) and/or aortic surgery) at the Department of Cardiothoracic Surgery of the LUMC from 1 January until 31 December 2017 were eligible to participate in the study. All patients participating gave written informed consent and filled out a self-developed survey. Patients who did not respond to the first written invitation, received a postal reminder after 2 and 4 weeks. Patients who received (partial) inpatient phase 2 CR were excluded from analysis.

### Assessments

A self-developed questionnaire was used to explore which factors were associated with referral to and enrolment in phase 2 CR. In addition, personal and disease characteristics were collected from patients’ medical files.

#### Survey

The development of the survey comprised the following steps. First, a list of factors that were found to contribute to referral to and enrolment in phase 2 CR were identified in studies that investigated phase 2 CR referral and enrolment of patients with cardiac disease. Subsequently, a panel of four experts (cardiothoracic surgeon, cardiologist, senior researcher/physical therapist and cardiothoracic physical therapist) rated which factors they considered most relevant. Finally, consensus was achieved for the eight most important and objectifiable factors. Five factors were included in the self-developed questionnaire. The three other factors were collected from the medical file. A concept version of the questionnaire was tested in five patients. Their feedback led to minor changes in the layout and wording of the survey.

The questionnaire consisted of the following topics to explore the potential factors that contributed to referral and enrolment: (1) educational level, (2) living status, (3) employment status (if < 67 years of age), (4) income status and (5) ethnicity. Additional questions regarding patient characteristics were aimed at smoking before surgery (yes/no, never smoked, quit smoking) and whether the patient was enrolled in phase 2 CR (yes/no).

#### Digital medical records

The following patient factors were collected from patients’ digital medical files: age, sex, type of open-heart surgery and length of hospital stay.

### Statistical analysis

Data collected from the questionnaire and from the patients’ medical files were entered into a database. Descriptive statistics (mean and standard deviation (SD), median and interquartile range (IQR) or number (percentage) when appropriate) were used to describe patient characteristics, self-reported referral to and enrolment in phase 2 CR, and potential factors related to referral and enrolment.

Differences in patient characteristics and potential factors between patients who were either referred or non-referred to phase 2 CR and between those who were enrolled or non-enrolled in phase 2 CR were compared using the chi-squared test (categorical variables), unpaired Student’s *t*-test or Mann-Whitney U‑test (numeric variables), when appropriate. *P*-values (two-tailed) < 0.05 were considered statistically significant.

Univariate logistic regression analysis was used to identify factors (age, sex, type of surgery, lower educational level (highest level was reference category), being single/having a partner, employment status (employed, voluntarily unemployed or involuntarily unemployed), lower income (highest income was reference category) and ethnicity) that were associated with non-enrolment or non-referral (both entered as dichotomous dependent variables). All independent variables were tested one-by-one in a univariate logistic regression analysis (*p* < 0.05). The logistic regression yielded an odds ratio with 95% confidence interval. Based on the rule of thumb that logistic models should be include a minimum of 10 outcome events per predictor variable, a sample size calculation was made [28, 29]. Since we identified eight potential factors, we estimated that (at least) 160 patients had to be included to allow for generalisation of the outcomes for the total group.

All data were analysed using IBM SPSS Statistics for Windows (version 24.0, Armonk, NY, USA).

## Results

**Fig. 2:**
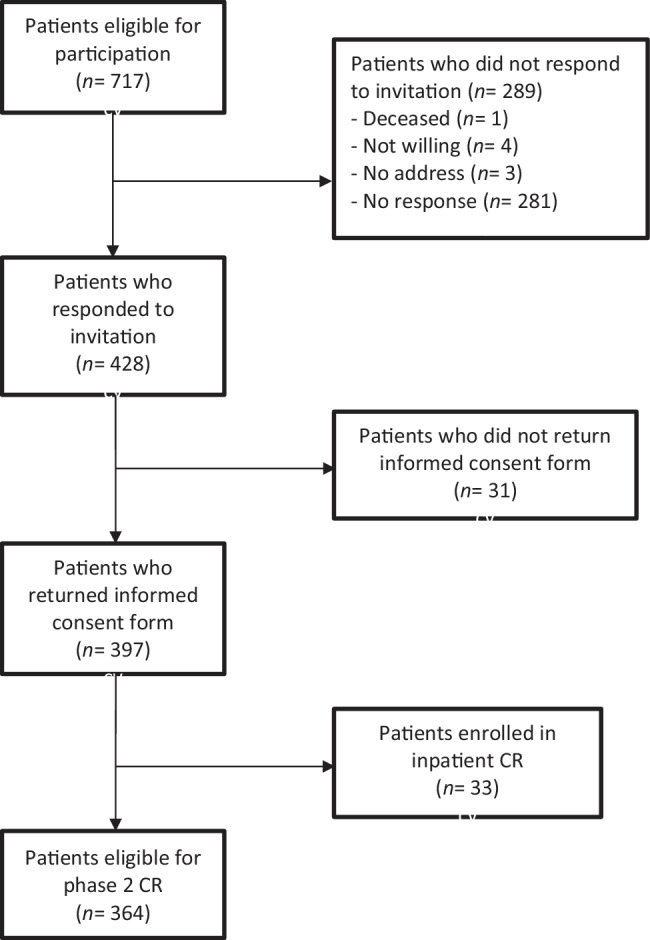
Flow chart of selection of patients invited to participate in the study. *CR* cardiac rehabilitation

A total of 397 patients (55%) out of 717 eligible patients returned a completed questionnaire and an informed consent form. Thirty-three patients were enrolled in inpatient CR instead of outpatient phase 2 CR, resulting in 364 (51%) responders who met the study inclusion criteria (Fig. 2).

### Patient characteristics

Tab. 1 shows the patient characteristics of the 364 respondents who were eligible for phase 2 CR. Of these 364 patients, 282 (78%) were male; the median age was 68.1 years (IQR 61.1–73.7). Most patients underwent CABG (162 patients, 45%) or valve surgery (72 patients, 20%). Twenty-nine (8%) patients underwent surgery that was not categorised because of the relatively small numbers, e.g. left ventricular assist device implantation or unroofing surgery (categorised as ‘other’, i.e. miscellaneous types of surgery). Patients stayed in the university hospital for a median duration of 9 days (IQR 7–12) before being transferred to another hospital or care facility or returning home.

**Table 1:** Characteristics of patients eligible for outpatient phase 2 cardiac rehabilitation following open-heart surgery via sternotomy at the Leiden University Medical Center in 2017

Variable	Patients (*N* = 364)
Age, years	68.1 (61.1–73.7)
Female sex	82 (22)
*Type of surgery*	
CABG	162 (45)
≥ 1 valve replacements or repairs	72 (20)
CABG combined with valve surgery	40 (11)
Aortic surgery	37 (10)
Aortic surgery, combined with CABG or valve surgery	24 (7)
Other	29 (8)
*Living status (n* *=* *361)*^a^	
Living alone	57 (16)
Not living alone	304 (84)
*Educational level (n* *=* *361)*^b^	
Low	142 (39)
Middle	94 (26)
High	125 (35)
*Employment status (<* *67 years of age) (n* *=* *165)*	
Employed	92 (56)
Voluntarily unemployed	47 (29)
Involuntarily unemployed	24 (15)
*Income per year (n* *=* *345)*^c^	
Below average	134 (39)
Average	99 (29)
Above average	112 (33)
*Born in the Netherlands (n* *=* *362)*	
Yes	316 (87)
No	46 (13)
*Parents born in the Netherlands (n* *=* *354)*	
Yes	302 (86)
No, both parents are born elsewhere	31 (9)
No, one parent is born elsewhere	21 (6)
Length of hospital stay, days	9 (7–12)
BMI, kg/m^2^ (median, IQR) (*n* = 361)	26.0 (23.7–28.6)
*Smoking (n* *=* *362)*^d^	
Current smoker	48 (13)
Previous smoker	198 (55)
Never smoker	116 (32)

*CABG* coronary artery bypass grafting, *BMI* body mass index

Data are median (interquartile range) or *n* (%)

^a^ Living status: living alone or not living alone (i.e. living with partner/family/others)

^b^ Educational level: ‘low’ is up to and including lower technical and vocational training; ‘middle’ is up to and including secondary technical and vocational training; ‘high’ is up to and including higher technical and vocational training and university

^c^ Income per year: ‘below average ’is < €37,000; ‘average’ is €37,000; ‘above average’ is > €37,000

^d^ Smoking before surgery: current smoker (answer option ‘yes’), previous smoker (‘quit smoking’) or never smoker (‘no’)

### Rates of referral and enrolment

The rate for referral to phase 2 CR was 84% and the enrolment rate was 87% (Tab. 2). Of the 47 patients who were not referred to phase 2 CR, 15 (32%) did eventually enrol in the programme. Of the 307 patients who were referred, 293 (95%) enrolled in phase 2 CR.

**Table 2:** Overview of referral to and enrolment in outpatient phase 2 cardiac rehabilitation (CR) of 364 patients following open-heart surgery

	Enrolled in phase 2 CR	Not enrolled in phase 2 CR	Enrolment unclear	Total
Referred to phase 2 CR	293 (80)	13 (4)	1 (0)	307 (84)
Not referred to phase 2 CR	15 (4)	31 (9)	1 (0)	47 (13)
Referral unclear if referred	7 (2)	3 (1)	–	10 (3)
Total	315 (87)	47 (13)	2 (1)	364 (100)

Data are *n* (%)

### Factors determining referral and enrolment

Tab. 3 shows several personal and disease characteristics of the referred and non-referred patients and of the enrolled and non-enrolled patients. In addition, Tab. 3 shows the odds ratios for non-referral to and non-enrolment in phase 2 CR. Female sex and older age were associated with both non-referral and non-enrolment. Moreover, living alone or having undergone CABG combined with valve surgery or miscellaneous types of relatively rare surgery were also associated with non-enrolment.

**Table 3:** Rates of referral to and enrolment in phase 2 cardiac rehabilitation and (sociodemographic, disease-related and environmental) factors associated with non-referral and non-enrolment of 364 patients following open-heart surgery

Factor	Non-referral		Referral		OR	95% CI	Non-enrolment		Enrolment		OR	95% CI
Total referral and enrolment rates	47 (13)		307 (84)				47 (13)		315 (87)			
Age, years		72 (65–76)		67 (60–73)	1.05	1.01–1.09		73 (70–77)		67 (60–73)	1.09	1.05–1.14
Female sex		17 (36)		59 (19)	2.37	1.23–4.59		17 (36)		63 (20)	2.26	1.17–4.35
*Type of surgery*												
CABG (reference category)		15 (32)		144 (47)	–	–		11 (23)		150 (48)	–	–
≥ 1 valve replacements or repairs		9 (19)		61 (20)	2.21	0.95–5.12		9 (19)		63 (20)	2.34	0.93–5.92
CABG combined with valve surgery		8 (17)		32 (10)	2.53	0.93–6.88		10 (21)		29 (9)	4.77	1.77–12.82
Aortic surgery		5 (11)		29 (9)	1.36	0.36–5.12		5 (11)		32 (10)	1.14	0.24–5.54
Aortic surgery, combined with CABG or valve surgery		4 (9)		19 (6)	2.17	0.56–8.48		4 (9)		20 (6)	2.86	0.71–11.55
Other		6 (13)		22 (7)	2.71	0.87–8.42		8 (17)		21 (7)	5.56	1.88–16.43
*Educational level*												
Low		23 (49)		117 (39)	1.38	0.68–2.78		25 (53)		115 (37)	1.86	0.90–3.81
Middle		9 (19)		81 (27)	0.78	0.32–1.87		9 (19)		85 (27)	0.90	0.37–2.21
High (reference)		15 (32)		106 (35)	–			13 (28)		112 (36)	–	
*Living status*												
Living alone		10 (21)		47 (16)	1.47	0.69–3.16		14 (30)		43 (14)	2.64	1.31–5.34
Living with partner/family/other (reference)		37 (79)		257 (85)	–	–		33 (70)		269 (86)	–	
*Employment status (<* *67 years of age)*												
Employed (reference)		5 (39)		87 (59)	–			3 (43)		89 (57)	–	
Voluntarily unemployed		5 (39)		41 (28)	2.18	0.60–7.94		2 (29)		45 (29)	1.35	0.22–8.36
Involuntarily unemployed		3 (23)		20 (14)	2.61	0.58–11.83		2 (29)		22 (14)	2.70	0.42–17.14
*Income per year*												
Below average		23 (54)		107 (37)	1.97	0.92–4.26		22 (50)		110 (37)	2.62	1.12–6.16
Average		9 (21)		85 (29)	0.94	0.38–2.43		14 (32)		85 (28)	2.14	0.86–5.35
Above average (reference)		11 (26)		100 (34)	–			8 (18)		104 (35)	–	
Not born in the Netherlands		6 (13)		39 (13)	1.00	0.40–2.51		6 (13)		40 (13)	1.02	0.41–2.57

Data are *n* (%) or median (interquartile range)

*OR* odds ratio, *CI* confidence interval, *CABG* coronary artery bypass grafting

## Discussion

This cross-sectional survey study in a Dutch university hospital showed that rates for referral to and enrolment in phase 2 CR following open-heart surgery were high: 84% and 87%, respectively, in a group of 364 patients. Factors that were associated with both non-referral and non-enrolment were female sex and older age. In addition, non-enrolment in phase 2 CR was associated with type of surgery, living alone or a below average income.

The 84% referral rate among the participants in our study is high. This rate is well over the 59% referral rate that was seen in a Dutch cohort of CABG patients [13] and comparable with the highest reported referral rates (60–90%) of patients with cardiac illness in other European countries [11]. This may indicate that cardiologists follow the guidelines for referring patients to phase 2 CR with sufficient room for individual considerations to decide otherwise. Regarding these considerations, in our analyses, non-referral was associated with female sex and older age, which was also reported in another study [10]. However, since we only examined the role of a limited number of variables, it is conceivable that the most decisive factors in the process of referring a patient to phase 2 CR were not included in our model. Such factors may include the patient being a caregiver for a spouse or any other practical or emotional constraints interfering with a patient taking part in phase 2 CR. It must not be underestimated that, apart from the treatment itself, travelling to a rehabilitation centre twice a week is a considerable burden for some patients—for example being dependent on family to accompany them or having to take a taxi—and this could be a reason not to choose the optimal treatment option from a medical perspective.

There is an important role for the cardiologist and other professionals to identify barriers that prevent patients from enrolling in phase 2 CR and to facilitate them by removing these barriers. The role of doctors and healthcare professionals is also acknowledged in the reviews by Resurrección et al. and Astley et al. [10, 14], who described that explicit clinical recommendation by a professional is one of the strongest predictors for enrolling in phase 2 CR. Cardiologists, thoracic surgeons and other health professionals involved in the (after)care of open-heart surgery patients should be conscious of their role in referring patients for phase 2 CR. Patients should be informed about the long-term positive outcomes of phase 2 CR programmes while taking their personal situation and ambitions into account. Furthermore, patients should be followed-up regardless of their enrolment in phase 2 CR, preferably by a specialised nurse with close connections to the physician.

Referral (84%) and enrolment rates (87%) were high in comparison with the enrolment rate (59%) described in a previous Dutch study using a 2007 cohort [13]. However, it is difficult to compare these rates because of the cross-sectional nature of our survey study. Still, our high enrolment rate can be partially explained by the update of the national guidelines in 2011 [5].

Both non-referral and non-enrolment were associated with female sex and older age. Therefore, extra attention should be directed to these subgroups to facilitate them to enrol in phase 2 CR. As mentioned previously, more qualitative research is needed to identify which personal circumstances contribute to non-enrolment and how to overcome these barriers. For example, being female more than doubled the odds of not being referred to and not enrolling in phase 2 CR. This is important information for physicians who are involved in the referral process, but qualitative research should lead to a deeper understanding of the reasons women are less likely to enrol in phase 2 CR. This enables cardiologists to target specific barriers, such as caring for a partner or logistical problems. Cardiologists or specialised nurses can motivate women to attend phase 2 CR programmes and overcome specific barriers or provide suitable alternatives for phase 2 CR. Nevertheless, cardiologists and subgroups of patients may have very acceptable reasons for non-enrolment and achieving 100% referral and enrolment rates is therefore no goal in itself.

### Limitations

Our study has several limitations. First, the patients completed the survey 1–2 years after surgery, which could have caused recall bias, and most information on the factors was self-reported. Second, although the response rate was more than 50%, there may have been selection bias since a substantial proportion of patients did not respond and there was no information on referral and enrolment for this patient group. Based on the approval document we received from the Medical Ethics Review Committee, we could not compare responders with non-responders. Third, generalisation of the findings should be done with care since patients were included from one Dutch hospital only. Culture concerning and attitude towards CR may vary by region or by country.

## Conclusion

This is the first study describing the rates of referral to and enrolment in phase 2 following open-heart surgery via sternotomy CR and exploring factors associated with non-referral and non-enrolment. In the present study, referral and enrolment rates for patients following open-heart surgery in a Dutch hospital were high. Nevertheless, several sociodemographic, disease-related and environmental factors were identified that were associated with non-referral to and non-enrolment in phase 2 CR.

Clearly, more (qualitative) research into subgroups of future patients and their reasons for not enrolling in phase 2 CR is recommended. Although there are some indications of subgroups of patients who are at risk of non-enrolment, a good conversation with the patient is essential for a better understanding of his or her deeper motives and capabilities for starting phase 2 CR. Possible barriers to non-enrolment should be eliminated with the support of the entire cardiology team if necessary. This way, all eligible patients have the opportunity to benefit from high-quality phase 2 CR and its long-term benefits.
